# Anticancer Potential of Biogenic Silver Nanoparticles: A Mechanistic Study

**DOI:** 10.3390/pharmaceutics13050707

**Published:** 2021-05-12

**Authors:** Mohd Shahnawaz Khan, Alya Alomari, Shams Tabrez, Iftekhar Hassan, Rizwan Wahab, Sheraz Ahmad Bhat, Nouf Omar Alafaleq, Nojood Altwaijry, Gouse M. Shaik, Syed Kashif Zaidi, Wessam Nouh, Majed S. Alokail, Mohamed A. Ismael

**Affiliations:** 1 Protein Research Chair, Department of Biochemistry, College of Science, King Saud University, Riyadh 11451, Saudi Arabia; alya.alomari7@gmail.com (A.A.); nalafaleg@ksu.edu.sa (N.O.A.); nojood@ksu.edu.sa (N.A.); gshaik@KSU.EDU.SA (G.M.S.); malokail@ksu.edu.sa (M.S.A.); mismael@ksu.edu.sa (M.A.I.); 2 King Fahd Medical Research Center, King Abdulaziz University, Jeddah 21589, Saudi Arabia; stabrez@kau.edu.sa; 3 Department of Medical Laboratory Technology, Faculty of Applied Medical Sciences, King Abdulaziz University, Jeddah 21589, Saudi Arabia; 4 Department of Zoology, College of Science, King Saud University, Riyadh 11451, Saudi Arabia; ihassan@ksu.edu.sa (I.H.); rwahab@ksu.edu.sa (R.W.); 5 Department of Biochemistry, New Science Block, SP College, Cluster University, Srinagar, Jammu and Kashmir 190008, India; sherazbhat@gmail.com; 6 Center of Excellence in Genomic Medicine Research, King Abdulaziz University, Jeddah 21589, Saudi Arabia; skashef@kau.edu.sa; 7 Department of Biochemistry, Faculty of Science, King Abdulaziz University, Jeddah 21589, Saudi Arabia; wnouh0001@stu.kau.edu.sa

**Keywords:** cytotoxicity, ROS, RT-PCR, scratch assay, silver nanoparticles

## Abstract

The continuous loss of human life due to the paucity of effective drugs against different forms of cancer demands a better/noble therapeutic approach. One possible way could be the use of nanostructures-based treatment methods. In the current piece of work, we have synthesized silver nanoparticles (AgNPs) using plant (*Heliotropium* *bacciferum)* extract using AgNO3 as starting materials. The size, shape, and structure of synthesized AgNPs were confirmed by various spectroscopy and microscopic techniques. The average size of biosynthesized AgNPs was found to be in the range of 15 nm. The anticancer potential of these AgNPs was evaluated by a battery of tests such as MTT, scratch, and comet assays in breast (MCF-7) and colorectal (HCT-116) cancer models. The toxicity of AgNPs towards cancer cells was confirmed by the expression pattern of apoptotic (p53, Bax, caspase-3) and antiapoptotic (BCl-2) genes by RT-PCR. The cell viability assay showed an IC_50_ value of 5.44 and 9.54 µg/mL for AgNPs in MCF-7 and HCT-116 cell lines respectively. We also observed cell migration inhibiting potential of AgNPs in a concentration-dependent manner in MCF-7 cell lines. A tremendous rise (150–250%) in the production of ROS was observed as a result of AgNPs treatment compared with control. Moreover, the RT-PCR results indicated the difference in expression levels of pro/antiapoptotic proteins in both cancer cells. All these results indicate that cell death observed by us is mediated by ROS production, which might have altered the cellular redox status. Collectively, we report the antimetastasis potential of biogenic synthesized AgNPs against breast and colorectal cancers. The biogenic synthesis of AgNPs seems to be a promising anticancer therapy with greater efficacy against the studied cell lines.

## 1. Introduction

Cancer and its associated secondary complications are one of the leading causes of global mortality because of the paucity of effective treatment, poor prognosis, and severe side effects of the existing chemotherapeutic drugs [1,2]. Among different types, breast cancer has been the most frequently reported cancer worldwide (13.7%), followed by colorectal cancer (11%). The existing chemotherapies for these two leading cancers often lead to secondary complications such as infections by bacteria, fungi, and viruses, as the immune system of these patients is vastly compromised. There is a wide range of cytotoxic drugs used for the treatment of breast cancer, such as doxorubicin, cisplatin, and bleomycin; they all have shown drawbacks and are inefficient [3]. Nanomedicine concerns the use of precision-engineered nanomaterials to develop novel therapeutic and diagnostic modalities for human use [4,5]. The convergence of nanotechnology and medicine has opened new avenues in therapeutic and pharmaceutical science [6]. Nanoparticles could act as anticancer nanomedicines used to target and treat cancerous cells [7].

The NPs could act as molecular probes [8], antiangiogenic [9], antitumor [10], antipermeability [11], and antiproliferative [6]. Among the nanomaterials, AgNPs have been reported to have the highest degree of commercialization and acquired repute in various fields, such as medicine and materials science [12]. AgNPs have special properties that empower these nanomedicines to oversee different pathological conditions successfully [13,14,15,16]. Because of their antiviral properties, AgNPs are compelling against hepatitis B [17], respiratory syncytial infection [18], herpes simplex infection type 1 [19], and monkeypox infection [20].

*Heliotropium bacciferum* is a perennial herb, growing in the Arabian Peninsula, where it is traditionally used against skin diseases and tonsillitis. It is a potent source of various bioactive phytochemicals. It is reported to have significant free radical scavenging activities in addition to antimicrobial, antihyperlipidemic, antidiabetic, and antitumor properties [21,22].

In the current piece of work, we have biologically synthesized cost-effective, rapid, and nontoxic AgNPs. The newly synthesized AgNPs were evaluated for their anticancer potential by using a battery of anticancer test such as cell viability, migration, cell morphology, ROS generation, and DNA damage among others in human breast cancer cells (MCF-7) and colon cancer cells (HCT-116).

## 2. Materials and Methods

### 2.1. Materials

MTT (3-(4,5-dimethylthiazol-2-yl)-2,5-diphenyltetrazoliumbromide), and 2,7 dichlorofluorescin diacetate (DCFH-DA) were obtained from Sigma-Aldrich (St. Louis, MO, USA). Fetal bovine serum (FBS), Dulbecco’s modified eagle’s medium (DMEM) were procured from Invitrogen Co., (Waltham, MA, USA). All other chemicals used were of the highest purity grade available from different commercial sources.

### 2.2. Biosynthesis of Metallic Nanoparticles

#### 2.2.1. Extract Preparation

*Heliotropium bacciferum* plant is a native to Saudi Arabia and was collected from Aljouf region (Kingdom of Saudi Arabia). A taxonomist in the Department of Botany, King Saud, University, Riyadh, KSA, confirmed the identity of this plant. The collected plant was further dried at room temperature under shade for 10 days. Thereafter, it was pulverized by mechanical grinder and stored in well-closed glassware containers until usage. For extraction, the coarse powder (250 gm) was soaked in 1-liter double distilled water and heated for 30 min at 60 °C under constant stirring. Next, the suspension was filtered using Whatman No. 1 filter paper, and the filtrate was dried using freeze dryer (Labconco, Kansas, MO, USA). For the total weight of *Heliotropium bacciferum*, extracted in water, the percentage yield was 36%. The residue was weighed and stored in a dark glass bottle at 4 °C for further experiments.

#### 2.2.2. Biosynthesis of Silver Nanoparticles

The freshly prepared plant extract (~5 mL) was mixed with AgNO_3_ (8 × 10^−3^ M, 45 mL) solution for the reduction of silver ions from the solution in a 100 mL beaker at room temperature. The reaction mixture was transferred to the black chamber, where no light could enter, and heated at 50 °C for 45 min. The solution was kept in dark until the color changed to brownish, which indicates the formation of AgNPs in the solution. The aqueous reduced silver nanoparticles solution was monitored on various instruments and was kept frozen at 4 °C for further examination.

### 2.3. Characterization of Synthesized Silver Nanoparticles

The morphology of synthesized AgNPs was analyzed by scanning electron microscopy (SEM, Jeol, JED-2200 series, Japan) at room temperatures. For this, the dried sample was sprayed on carbon tape and pasted on the sample holder. The sample holder was transferred to the glass chamber where the sputtering was completed for 2–3 s. Once the sample preparation was completed, it was fixed with the sample holder and analyzed at room temperature. Further, the morphology of biosynthesized nanostructure was also confirmed by transmission electron microscopy (TEM) (JEOL, JSM 2010, Tokyo, Japan). For this, the colloidal sample was sonicated for about 10–15 min in a bath sonicator (~40 kHz, Cole Parmer, IL, USA) and then after a carbon-coated copper grid (~400 mesh size) was dipped to this solution and dried at room temperature. After the complete drying, the copper grid was fixed to the sample holder and analyzed at room temperature.

The chemical functional bonding of synthesized AgNPs was accessed by Fourier-transform infrared spectroscopy (FTIR) (Perkin Elmer’s GX spectrophotometer). The powder liquid sample was mixed with potassium bromide powder (KBr), and a pellet was formed with pressure (4 *tons*) and this pellet was fixed to the sample holder and analyzed in the range of 400–4000 cm^−1^. The crystallinity of the material, crystallite size, phases, and full-width half maxima (FWHM) of the sample was accessed by an X-ray powder diffractometer (XRD) with Cu_Kα_ radiation (λ = 1.54178Å) in the range of 20–85º with 6º/min scanning speed at 40 mV and 30 kV.

### 2.4. Cytotoxicity/Viability Measurement (MTT Assay)

The cytotoxicity of AgNPs was measured by 3-(4,5-dimethylthiazol-2-yl)- 2,5-diphenyltetrazolium bromide dye reduction assay. Briefly, the MCF-7 and HCT-116 cells were plated on 96-well flat-bottom culture plates and treated with different concentrations of AgNPs. All cultures were incubated at 37 °C for 24 h in a humidified incubator. After 24 h of incubation, 10 µL of MTT (5 mg/mL stock in PBS) was added to each well, and the plates were incubated for 4 h at 37 °C. The resulting formazan was dissolved in 100µL of dimethyl sulfoxide (DMSO) with gentle shaking at 37 °C, and absorbance was measured at 460 nm on an ELISA plate reader (Spectra MAX; Molecular Devices, San Jose, CA, USA). At least three independent experiment sets were performed, and results were calculated as the mean of the three independent values. Concentrations of AgNPs showing a 50% reduction in cell viability (IC_50_) were calculated with the help of Prism software.

### 2.5. Cell Migration (Scratch Assay)

To observe the effect of AgNPs on the cell–cell interaction and cell migration, we performed the in vitro scratch assay [23]. The images of control and test wells (treated with 3 mg/mL AgNPs) were taken at 0, 24, and 48 h.

### 2.6. ROS Measurement

Intracellular reactive oxygen species (ROS) were estimated based on the intracellular peroxide-dependent oxidation of 2,7-164 dichlorodihydrofluorescein diacetate, as described earlier [24]. Cells were seeded onto 24-well plates at a density of 5 × 10^4^ cells/well. After washing twice with PBS, a fresh medium containing 17.4 µM of AgNPs or 1 mM H_2_O_2_ was added and the treated cells were incubated for 24 h. For control, 20 µM of DCFH-DA was added to the cells and incubated for 30 min at 37 °C. The cells were rinsed with PBS, and fluorescence intensity was measured on a spectrofluorometer (Gemini EM) with excitation and emission spectra at 485 nm and 530 nm, respectively. For control, the 24 h grown cells were added with a well-known antioxidant N-acetyl-L-cystein (NAC, 5 mM) 1 h before treatment.

### 2.7. Gene Expression Analysis (Real Time-PCR)

To measure the expression pattern of certain pro/anti apoptotic genes, RT-PCR was performed by QuantiTect SYBR green PCR kit (Qiagen) using an ABI PRISM 7900HT sequence detection system (Applied Biosystems, Foster City, CA, U.S.A). Real-time PCR cycle parameters included 10 min initial denaturation at 95 °C followed by 40 cycles involving denaturation at 95 °C for 15 s, annealing at 60 °C for 20 s, and elongation at 72 °C for 20 s. The sequences of the specific sets of primer for various apoptotic genes such as bax, bcl-2, caspase-3, and p53 used in this study have been taken as reported elsewhere [25]. The real-time PCR experiments were performed in triplicate and data expressed as the mean of at least three independent experiments.

### 2.8. Comet Assay

The comet assay was conducted as per the method described by Singh [26] with some modifications tailored for cell lines [27]. The fully grown cells were treated with different concentrations of AgNPs for 3 h in different petri dishes (60 × 15 mm, Greiner) in the suitable media and conditions. After that, the cells were trypsinized for the preparation of cell suspension followed by homogenization in 1 mL of the medium. The cells were then centrifuged at 500 g for 5 min. The cells suspension (100 μL) was added to 100 μL of 1% low melting agarose (Sigma-Aldrich, St Louis, MO, USA). After that, 100 μL of this solution was layered on the normal melting agarose (Merck, Kenilworth, NJ, USA) base coated slides in duplicates for each group. The slides were kept in the lysis buffer (0.045 M TBE, pH 8.4, containing 2.5% SDS) for 10 min. Then, the slides were placed in a comet assay tank having the same TBE buffer for 10 min without SDS at the voltage and current strength of 2 V/cm and 80 mA respectively. Finally, ethidium bromide (20 μg/mL, Sigma-Aldrich, USA) was used to stain the cells. After washing delicately, the slides were covered with the coverslips (Blue Star, Mumbai, India) and stored in humid conditions. The tail-length of nuclear DNA of 100 cells for each group was assessed with an upright fluorescent microscope (Leica DM2500, Bensheim, Germany) attached with a digital CCD camera). The measurement of tail length and imaging was conducted by Komet 5.5 image analysis software.

### 2.9. Statistical Analysis

The data obtained from each experiment were presented as mean ± standard error values of three independent replicates. The difference between control and test was analyzed using the Student’s t-test or otherwise stated in their respective legends.

## 3. Results and Discussion

### 3.1. Biosynthesis of Silver Nanoparticles (AgNPs)

**Figure 1:**
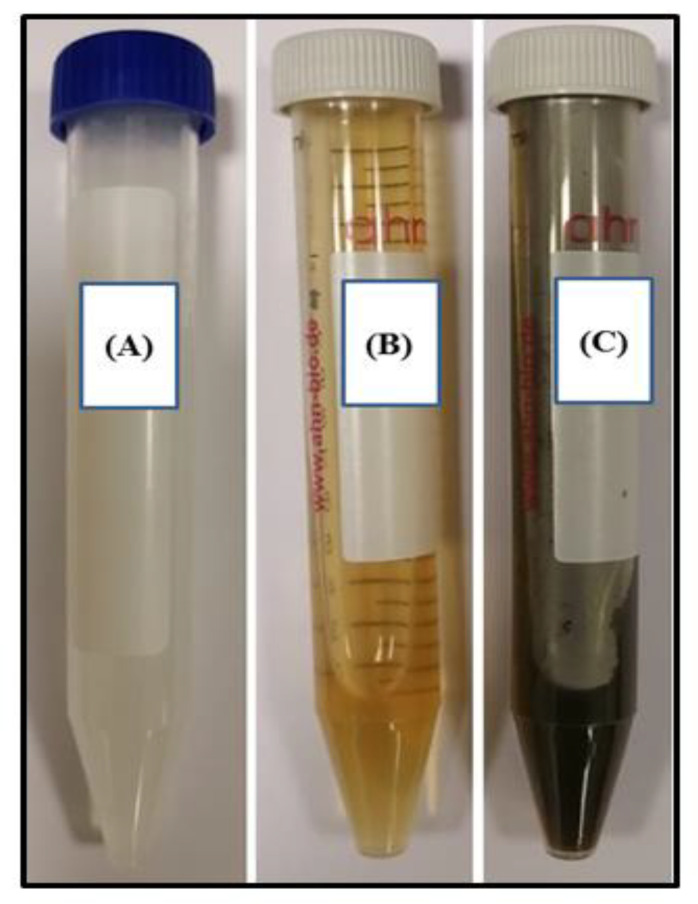
The solution of AgNO_3_ (**A**)_,_ after reaction with plant extract (**B**), synthesized silver nanoparticles (AgNPs) (**C**).

Figure 1A shows the image of silver nitrate and illustrates that there is no oxidation or reduction of silver particles. Once mixed with the plant extract, a yellowish color solution was formed (Figure 1B). The reaction tube was completely covered with aluminum foil and kept in the oven for 40–45 min at 50 °C. The yellowish color solution changed to a dark brown color, which indicates the reduction of silver nitrate salt into silver ions in the presence of processed extract. The change in color indicated the formation of AgNPs (Figure 1C).

### 3.2. Characterization of Synthesized Silver Nanoparticles by a Multitechnique Approach

**Figure 2:**
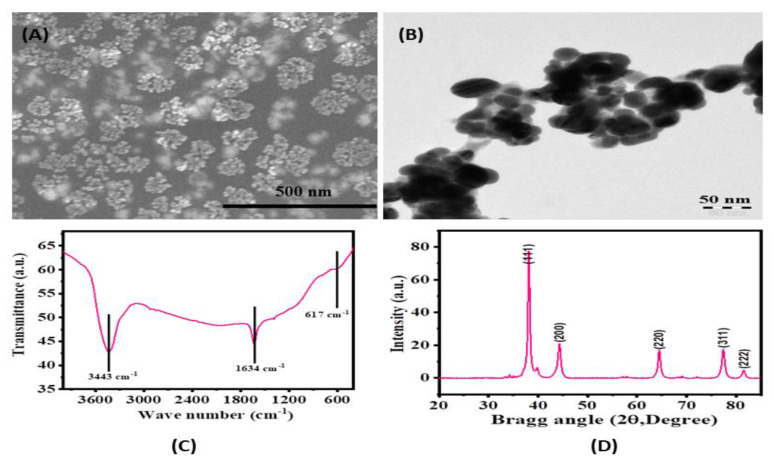
(**A**): Scanning electron microscopy (SEM) image of biogenic silver nanoparticles (size ~15 nm), (**B**) Transmission electron microscopy (TEM) image of prepared silver nanoparticles from the extract (average size ~15 nm). (**C**) Fourier-transform infrared (FTIR) spectra of silver nanoparticles showing the functional groups in used chemicals. (**D**) X-ray diffraction spectrum of biosynthesized silver nanoparticles.

Figure 2A illustrates the SEM image of the green synthesized (with plant extract) silver nanoparticles (AgNPs). The acquired data show several small sizes, smooth surfaces that are either spherical or oval-shaped structures. The small particles were found to be either aggregated or clumped with other particles. From the detailed observation, it reveals that the average size of each NP was in the range of 15 nm.

The morphological examination was done by transmission electron microscopy (TEM) at room temperature as described in the material and methods. The obtained image (Figure 2B), illustrates that the recovered NPs are spherical with smooth surfaces and have good morphological characteristics. The average size of each NP was in the range of 14 ± 1 nm, aggregated, which is analogous to the X-ray diffraction data (Figure 2C).

The functional footprint of the biosynthesized AgNPs was examined by FTIR spectroscopy (Figure 2D). The bands at 3433 cm^−1^, 1634 cm^−1^, and 617 cm^−1^, revealed the involved components in the formation of AgNPs. The peaks at 3443 cm^−1^ and 1634 cm^−1^ indicate the stretching and bending mode of the O–H bond, respectively. The peak assigns at 617 cm^−1^ indicated the silver metal peak as reported earlier too [28,29]. The FTIR data suggest that the AgNPs were synthesized without any capping agent.

### 3.3. AntcCancer Activity of AgNPs: MTT, Scratch, and Comet Assay

**Figure 3:**
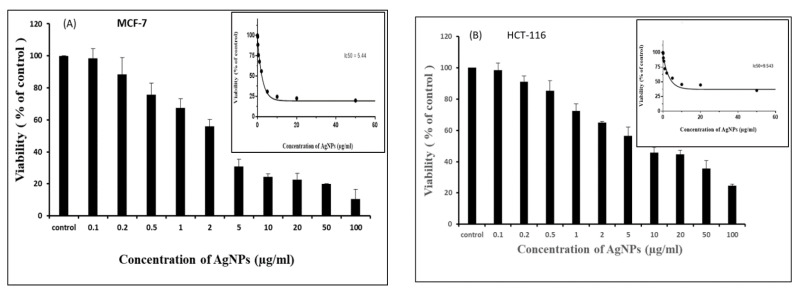
Cell viability measurement by MTT-based colorimetric method. Cells were treated with different concentrations of AgNPs for 24 h. The results represent the means of three separate experiments for each MCF-7 (**A**) and HCT-116 (**B**) cell lines and error bars represent the standard error of the mean. Treated groups showed statistically significant differences from the control group by ANOVA (*p* < 0.05). IC_50_ values were measured by prism software (Inset).

**Figure 4:**
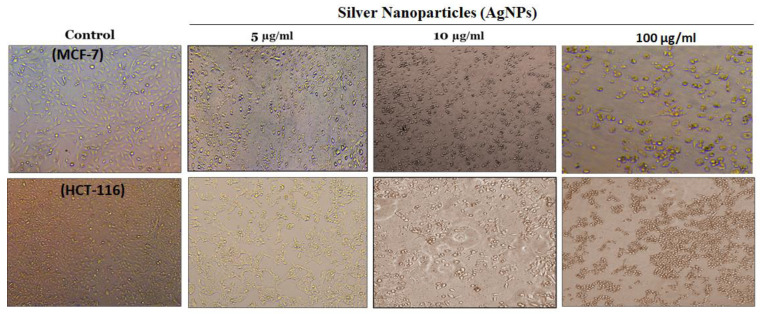
Morphological changes in MCF-7 and HCT-116 cells treated with increasing concentration of AgNPs after 24 h. Leica microscope having 10× magnification was used to capture the images.

After confirming the quality of the biosynthesized AgNPs, the biological features of the AgNPs were assessed by a battery of tests. Cell viability was measured by a colorimetric method based on MTT [3-(4,5-dimethylthiazol-2-yl)-2,5-diphenyl tetrazolium bromide] reduction (Figure 3A,B). The AgNPs were found to be identically cytotoxic towards MCF-7 and HCT-116 cell lines in a concentration-dependent manner with an IC_50_ value of 5.44 and 9.54 µg/mL, respectively. Morphological changes were observed after treatment with AgNPs in both cell types (Figure 4). The cancer cells become distorted and lose their morphology with increasing concentration of biosynthesized AgNPs. The cytotoxicity of AgNPs is well-established and depends on the nature of cell types and the size of NPs [29]. Cytotoxicity of AgNPs has been reported in many cancer cell lines such as human Chang liver cells and rat basophil leukemia (RBL) cells [30]. The AgNPs have also been reported to be more toxic toward cancer cells compared to normal cells [31,32,33,34]. The cytotoxicity of NPs depends on their size and shape, which in turn varies with the method of their preparation. It also depends on the types of cancer cells that have abnormal metabolism and morphology [35].

**Figure 5:**
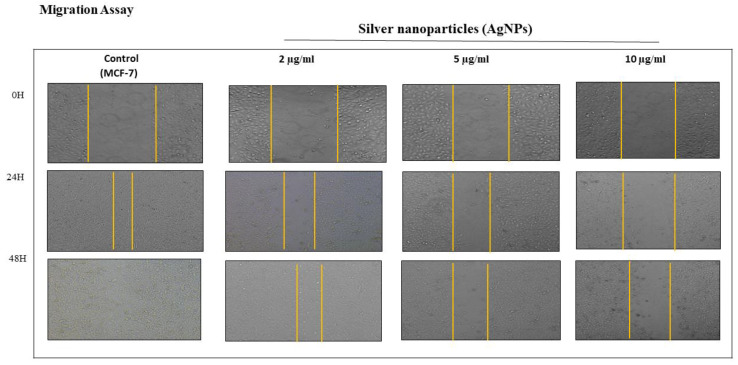
Scratch was made onto a monolayer of each cell line and treated by 2–10 µg/mL of AgNPs (indicated appropriately in the figure) for different time points. Comparison of migration in both control and test was made by taking images at different time intervals (0, 24, and 48 h). Results are represented by marking the scratch with parallel lines and visually displaying the number of cells migrated to the scratch area denoting metastasis inhibition.

Apart from uncontrolled divisions, cancer has the peculiar feature of invading other tissues by metastasis, that is, cell–cell migration. In the present study, the effect of AgNPs on cell migration was studied by scratch assay [23]. We observed effective cell migration inhibiting potential of AgNPs in a concentration-dependent manner by scratch plate assay in MCF-7 cell lines (Figure 5).

AgNPs showed a significant reduction in cell migration at 2 µg/mL in MCF-7 cells after 48 h of incubation compared to control. At further higher concentrations of AgNPs, higher inhibition was observed, which was found to be variable with time (Figure 5). The migration pattern of HCT-116 cells could not be measured due to their growth pattern in bunches (data not shown). The inhibition of migration of MCF-7 by AgNPs could be due to the ability of AgNPs to interfere with the cell’s cytoskeleton. Cell division and migration require cytoskeleton rearrangement, and interference in one or both processes shows a notable effect on cell proliferation and migration. Our results are in good agreement with earlier studies [36,37].

**Figure 6:**
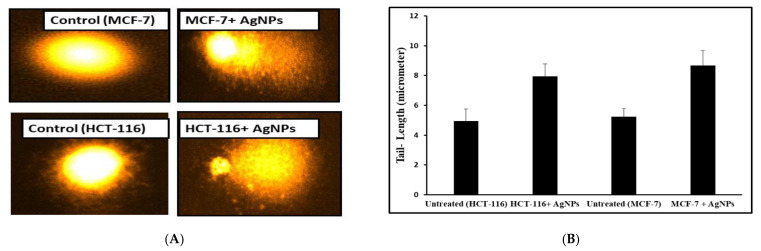
Comet assay to assess DNA damage induced by AgNPs in cancer cells. The results demonstrated significant DNA damage by AgNPs (pictograph, **A**) and as evident by the tail length of DNA in cancer cells (**B**).

Comet assay measured a combination of single-strand breaks, double-strand breaks, and alkaline labile sites [38]. The present study showed a significant effect of AgNPs on the nuclear DNA of MCF-7 and HCT-116 cells. AgNPs were found to increase the comet tail by 65.64% and 60.60% in MCF-7 and HCT-116 cells, respectively, compared to the control cells (Figure 6A,B). This result indicates a slightly better anticancer potential of AgNPs in MCF-7 cells compared to HCT-116 cells. The observed result reaffirms the MTT data of cell viability where AgNPs showed lower IC_50_ value for MCF-7 than HCT-116.

### 3.4. Cellular Reactive Oxygen Species (ROS) Response of AgNPs

**Figure 7:**
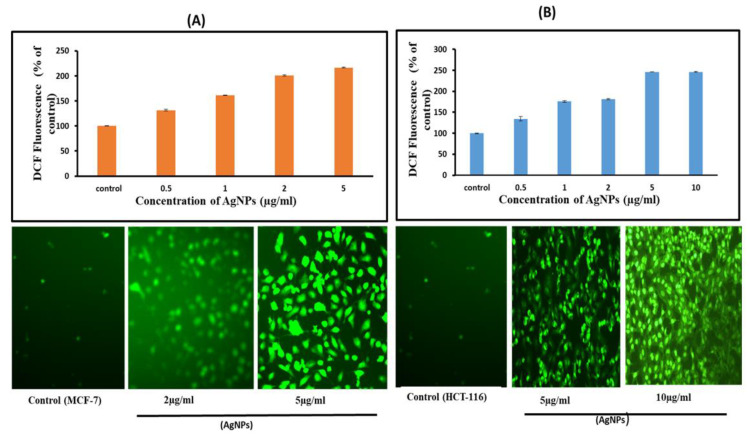
Oxidative stress response of AgNPs towards MCF-7 and HCT-116. ROS generation in AgNPs treated MCF-7 (**A**) and HCT-116 (**B**) cells. Relative fluorescence of DCF was measured using a spectrofluorometer with excitation at 485 and emission at 530 nm. The results represent the means of three separate experiments, ad error bars represent the standard error of the mean. Treated groups showed statistically significant differences from the control group by ANOVA (*p* < 0.05). Image of the cells showing fluorescence was captured by a Leica microscope (20 × magnification).

Oxidative stress is one of the key mechanisms of nanoparticles induced toxicity [39,40]. The interaction between AgNPs and mammalian cells can induce oxidative stress by inducing cellular ROS production over the cellular antioxidant defences [6]. The DCF fluorescence was measured in cancer cells treated with AgNPs at increasing concentrations from 2–10 µg/mL for 24 h. There was a tremendous rise in the production of ROS ranging from 150–250% compared to control, indicating the enormous ROS generating capability of AgNPs in MCF-7 cell lines (Figure 7A). An almost similar result was obtained in HCT-116 cells too (Figure 7B). The balance in redox potential in a physiological system is essential for normal biochemical functions, and abnormality in this balance causes oxidative stress resulting in pathological processes [40,41]. The overproduction of ROS in the cells is known to induce apoptosis. ROS generation has appeared to assume a significant part in apoptosis induced by treatment with AgNPs [6,41,42]. Prior investigations showed that AgNPs could incite the production of ROS by macrophages and human Chang liver cells [29,43].

Our study provided evidence for a molecular mechanism of AgNPs-induced generation of ROS that could induce apoptosis. Taken together, all these results indicate that cell death observed by us is mediated by ROS production, which might have altered the cellular redox status.

### 3.5. AgNPs Altered the Expression of mRNA and Protein Levels of Apoptotic Genes

**Figure 8:**
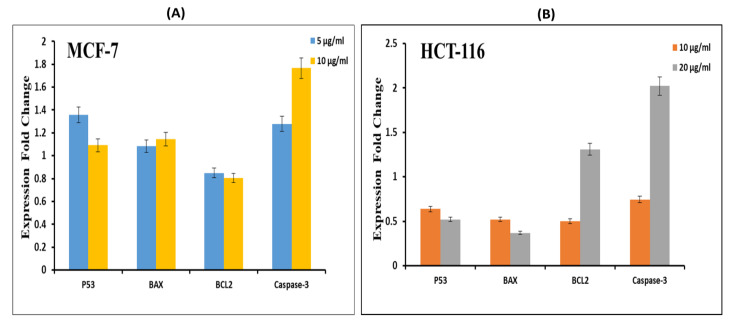
Effect of AgNPs on mRNA expression level of apoptotic markers in cancer cells. Cells were treated with different concentration of AgNPs for 24 h. Nanostructure-induced alterations in mRNA expression levels are expressed as fold change in relative quantity with those of control cells. CT values for other genes were calculated in reference to GAPDH control. (**A**) Breast cancer cells (MCF-7) were treated with 5 µg/mL and 10 µg/mL silver nanoparticles, while (**B**) HCT-116 cells were incubated with 10 and 20 µg/mL for 24 h.

Quantitative real-time PCR was used to analyze the mRNA levels of apoptotic genes (p53, bax, bcl-2, and caspase-3) in cancer cells exposed to different concentrations (10 and 20 μg/mL) of AgNPs for 24 h. Results showed that AgNPs significantly altered the expression levels of mRNA of these genes in both the cell lines (Figure 8). The mRNA expression level of tumor suppressor gene p53 and pro-apoptotic gene bax was found to be significantly upregulated while the expression of antiapoptotic gene bcl-2 was found to be significantly downregulated in AgNP treated MCF-7 and HCT-116 cells (Figure 8A,B) compared to control cells. We also observed higher level of caspase-3, mRNA in AgNP-treated cells than those of control cells (Figure 8). 

In the presence of DNA damage or cellular stress, p53 triggers cell-cycle arrest to offer time for the damage to be repaired or for self-mediated apoptosis. Activated caspase-3 is fit for autocatalysis as well as cleaving and activating other members of the caspase family, resulting in quick and irreversible apoptosis. In the present study, we also observed the DNA fragmentation and higher activity of caspase-3 enzyme in MCF-7 and HCT-116 cancer cells treated with AgNPs. Overall, the RT-PCR results indicated the difference in expression levels of studied apoptotic proteins in both the cancer cell lines highlighting the anticancer potential of our biosynthesized AgNPs, given the specific apoptotic response in cancer cells.

## 4. Conclusions

In the current study, we have found the distinctive effects of biogenic AgNPs on mammalian cell viability of cancer cells (HCT-116, and MCF-7). The marked difference in AgNP induced cytotoxicity in cancer cells indicates a possible alternative to cancer treatment over the conventional methods, which have higher side effects. Our molecular data revealed up/downregulated levels of pro/antiapoptotic in both cell lines treated with AgNPs, suggesting a tumor-suppression mechanism. Moreover, AgNPs induced DNA fragmentation and also altered cellular redox status in HCT-116 and MCF-7 cells. This suggests that AgNPs induce apoptosis in cancer cells, which is mediated by ROS via p53, bax/bcl-2, and caspase pathways. Based on the specific apoptotic response, the current nanostructure showed much promise as an anticancer agent. However, more elaborate studies are warranted to determine the exact difference that resulted in cancer cell-specific toxicity of AgNPs, which is still largely unclear.

## Data Availability

Not applicable.

## References

[1] Siegel R.L., Miller K.D., Jemal A. (2017). Cancer statistics, 2017. CA Cancer J. Clin..

[2] Islam B., Khan M.S., Husain F., Rehman M.T., Alzughaibi T., Abuzenadah A.M., Urooj M., Kamal M.A., Tabrez S. (2020). mTOR targeted cancer chemoprevention by flavonoids. Curr. Med. Chem..

[3] Park Y.H., Hwang C., Kim Y., Lee Y., Jeong D., Cho M. (2007). Antimicrobial effects of silver nanoparticles. Nanomedicine.

[4] Tabrez S., Jabir N.R., Adhami V.M., Khan M.I., Moulay M., Kamal M.A., Mukhtar H. (2020). Nanoencapsulated dietary polyphenols for cancer prevention and treatment: Successes and Challenges. Nanomedicine (Lond.).

[5] Sriram M.I., Mani Kanth S.B., Kalishwaralal K., Gurunathan S. (2010). Antitumor activity of silver nanoparticles in Dalton’s lymphoma ascites tumor model. Int. J. Nanomed..

[6] Asha Rani P.V., Hande M.P., Valiyaveettil S. (2009). Anti-proliferative activity of silver nanoparticles. BMC Cell Biol..

[7] Oves M., Aslam M., Rauf M.A., Qayyum S., Qari H.A., Khan M.S. (2018). Antimicrobial and anticancer activities of silver nanoparticles synthesized from the root hair extract of *Phoenix dactylifera*. Mater. Sci. Eng. C.

[8] Lok C., Ho C., Chen R., He Q., Yu W., Sun H., Tam P., Chiu J., Che C. (2007). Silver nanoparticles: Partial oxidation and antibacterial activities. J. Biol. Inorg. Chem..

[9] Gurunathan S., Lee K.J., Kalishwaralal K., Sheikpranbabu S., Vaidyanathan R., Eom S.H. (2009). Antiangiogenic properties of silver nanoparticles. Biomaterials.

[10] Franco-Molina M.A., Gamboa E.M., Sierra-Rivera C.A., Gómez-Flores R.A., Zapata-Benavides P., Castillo-Tello P., Alcocer-González J.M., Miranda-Hernández D.F., Tamez-Guerra R.S., Rodríguez-Padilla C. (2010). Antitumor activity of colloidal silver on MCF-7 human breast cancer cells. J. Exp. Clin. Cancer Res..

[11] Sheikpranbabu S., Kalishwaralal K., Venkataraman D., Eom S.H., Park J., Gurunathan S. (2009). Silver nanoparticles inhibit VEGF-and IL-1beta-induced vascular permeability via Src dependent pathway in porcine retinal endothelial cells. J. Nanobiotechnol..

[12] Ahmed M., Karns M., Goodson M., Rowe J., Hussain S., Schlager J., Hong Y. (2008). DNA damage response to different surface chemistry of silver nanoparticles in mammalian cells. Toxicol. Appl. Pharmacol..

[13] Bhattacharya R., Mukherjee P. (2008). Biological properties of “naked” metal nanoparticles. Adv. Drug Deliv. Rev..

[14] Kalishwaralal K., Deepak V., Pandian S.R.K., Kottaisamy M., BarathManiKanth S., Kartikeyan B., Gurunathan S. (2010). Biosynthesis of silver and gold nanoparticles using Brevibacterium casei. Colloid Surface B Biointerfaces.

[15] Sun R.W., Rong C., Chung N.P.Y., Ho C.M., Lin C.L.S., Che C.M. (2005). Silver nanoparticles fabricated in Hepes buffer exhibit cytoprotective activities toward HIV-1 infected cells. Chem. Commun..

[16] Lara H.H., Nuñez N.V.A., Turrent L.I., Padilla C.R. (2010). Mode of antiviral action of silver nanoparticles against HIV-1. J. Nanobiotechnol..

[17] Lu L., Sun R.W., Chen R., Hui C.K., Ho C.M., Luk J.M., Lau G.K., Che C.M. (2008). Silver nanoparticles inhibit hepatitis B virus replication. Antivir. Ther..

[18] Morris D., Ansar M., Speshock J., Ivanciuc Y., Qu Y., Casola A., Garofalo R.P. (2019). Antiviral and Immunomodulatory Activity of Silver Nanoparticles in Experimental RSV Infection. Viruses.

[19] Baram-Pinto D., Shukla S., Perkas N., Gedanken A., Sarid R. (2009). Inhibition of herpes simplex virus type 1 infection by silver nanoparticles capped with mercaptoethane sulfonate. Bioconjug. Chem..

[20] Rogers J.V., Parkinson C.V., Choi Y.W., Speshock J.L., Hussain S.M. (2008). A Preliminary Assessment of Silver Nanoparticle Inhibition of Monkeypox Virus Plaque Formation. Nanoscale Res. Lett..

[21] Ahmad S., Ahmad S., Bibi A., Ishaq M.S., Afridi M.S., Kanwal F., Zakir M., Fatima F. (2014). Phytochemical Analysis, Antioxidant Activity, Fatty Acids Composition, and Functional Group Analysis of *Heliotropium bacciferum*. Sci. World J..

[22] Ahmad S., AbdEl-Salam N.M., Ullah R. (2016). In Vitro Antimicrobial Bioassays, DPPH Radical Scavenging Activity, and FTIR Spectroscopy Analysis of *Heliotropium bacciferum*. BioMed Res. Int..

[23] Liang C.C., Park A.Y., Guan J.L. (2007). In vitro scratch assay: A convenient and inexpensive method for analysis of cell migration in vitro. Nat. Protoc..

[24] Shim H.Y., Park J.H., Paik H.D., Nah S.Y., Kim D.S.H.L., Han Y.S. (2007). Acacetin-induced Apoptosis of Human Breast Cancer MCF-7 Cells Involves Caspase Cascade, Mitochondria-mediated Death Signaling and SAPK/JNK1/2-c-Jun Activation. Mol. Cells.

[25] Ahamed M., Akhtar M.J., Siddiqui M.A., Ahmad J., Musarrat J., Al-Khedhairy A.A., AlSalhi M.S., Alrokayan S.A. (2011). Oxidative stress mediated apoptosis induced by nickel ferrite nanoparticles in cultured A549 cells. Toxicology.

[26] Singh N.P., McCoy M.T., Tice R.R., Schneider E.L. (1988). A simple technique for quantization of low levels of DNA damage in individual cells. Exp. Cell. Res..

[27] Hassan I., Khan A.A., Aman S., Qamar W., Ebaid H., Al-Tamimi J., Alhazza I.M., Rady A.M. (2018). Restrained management of copper level enhances the antineoplastic activity of imatinib in vitro and in vivo. Sci. Rep..

[28] Kalpana D., Han J.H., Park W.S., Lee S.M., Wahab R., Lee Y.S. (2019). Green biosynthesis of silver nanoparticles using *Torreya nucifera* and their antibacterial activity. Arab. J. Chem..

[29] Park M.V., Neigh A.M., Vermeulen J.P., de la Fonteyne L.J., Verharen H.W., Briedé J.J., Van Loveren H., De Jong W.H. (2011). The effect of particle size on the cytotoxicity, inflammation, developmental toxicity and genotoxicity of silver nanoparticles. Biomaterials.

[30] Piao M.J., Kang K.A., Lee I.K., Kim H.S., Kim S., Choi J.Y., Choi J., Hyun J.W. (2011). Silver nanoparticles induce oxidative cell damage in human liver cells through inhibition of reduced glutathione and induction of mitochondria involved apoptosis. Toxicol. Lett..

[31] Gopinath P., Gogoi S.K., Chattopadhyay A., Ghosh S.S. (2008). Implications of silver nanoparticle induced cell apoptosis for in vitro gene therapy. Nanotechnology.

[32] Arora S., Jain J., Rajwade J.M., Paknikar K.M. (2009). Interactions of silver nanoparticles with primary mouse fibroblasts and liver cells. Toxicol. Appl. Pharmacol..

[33] Faedmaleki F., Shirazi F.H., Ejtemaeimehr S., Anjarani S., Salarian A.A., Ahmadi Ashtiani H., Rastegar H. (2016). Study of Silymarin and Vitamin E protective effects on silver nanoparticle toxicity on mice liver primary cell culture. Acta Med. Iran.

[34] Faedmaleki F., Shirazi F.H., Salarian A.A., Ashtiani H.A., Rastegar H. (2014). Toxicity effect of silver nanoparticles on mice liver primary cell culture and HepG2 cell line. Iran. J. Pharm. Res..

[35] Perde-Schrepler M., Florea A., Brie I., Virag P., Fischer-Fodor E., Angela Vâlcan A., Eugen Gurzău E., Cosmin Lisencu C., Maniu A. (2019). Size-Dependent Cytotoxicity and Genotoxicity of Silver Nanoparticles in Cochlear Cells In Vitro. J. Nanomater..

[36] Buranasukhon W., Athikomkulchai S., Tadtong S., Chittasupho C. (2017). Wound healing activity of *Pluchea indica* leaf extract in oral mucosal cell line and oral spray formulation containing nanoparticles of the extract. Pharm. Biol..

[37] Khan M.S., Tabrez S., Al-Okail M.S., Shaik G.M., Bhat S.A., Rehman M.T., Husain F.M., AlAjmi M.F. (2021). Non-enzymatic glycation of protein induces cancer cell proliferation and its inhibition by quercetin: Spectroscopic, cytotoxicity and molecular docking studies. J. Biomol. Struct. Dyn..

[38] Ali D., Ray R.S., Hans R.K. (2010). UVA-induced cytotoxicity and DNA damaging potential of benz (e) acephenanthrylene. Toxicolo. Lett..

[39] Foldbjerg R., Olesen P., Hougaard M., Dang D.A., Hoffmann H.J., Autrup H. (2009). PVP-coated silver nanoparticles and silver ions induce reactive oxygen species, apoptosis and necrosis in THP-1 monocytes. Toxicol. Lett..

[40] Sastre J., Pallardo F.V., Vina J. (2000). Mitochondrial oxidative stress plays a key role in aging and apoptosis. IUBMB Life.

[41] Martindale J.L., Holbrook N.J. (2002). Cellular response to oxidative stress: Signaling for suicide and survival. J. Cell. Physiol..

[42] Carlson C., Hussain S.M., Schrand A.M., Braydich-Stolle L.K., Hess K.L., Jones R.L. (2008). Unique cellular interaction of silver nanoparticles: Size-dependent generation of reactive oxygen species. J. Phys. Chem. B.

[43] Sánchez-Pérez Y., Chirino Y.I., Osornio-Vargas Á.R., Morales-Bárcenas R., Gutiérrez-Ruíz C., Vázquez-López I., García-Cuellar C.M. (2009). DNA damage response of A549 cells treated with particulate matter (PM10) of urban air pollutants. Cancer Lett..

